# Utilization of Neem Leaf Extract on Biosynthesis of Iron Oxide Nanoparticles

**DOI:** 10.3390/molecules24203803

**Published:** 2019-10-22

**Authors:** Nur Diyana Syazwani Zambri, Nurul Izza Taib, Famiza Abdul Latif, Zakiah Mohamed

**Affiliations:** 1 Faculty of Applied Sciences, Universiti Teknologi MARA, UiTM, Shah Alam 40450, Selangor, Malaysia; 2 Faculty of Applied Sciences, Universiti Teknologi MARA, Perak Branch, Tapah Campus, Tapah Road 35400, Perak, Malaysia

**Keywords:** biosynthesis, iron oxide nanoparticles, *Azadirachta indica*, superparamagnetic

## Abstract

The present work reports the successful synthesis of biosynthesized iron oxide nanoparticles (Fe_3_O_4_-NPs) with the use of non-toxic leaf extract of Neem (*Azadirachta indica)* as a reducing and stabilizing agent. The successful synthesis was confirmed by infrared spectra analysis with strong peak observed between 400–600 cm^−1^ that corresponds to magnetite nanoparticles characteristics. X-ray diffraction (XRD) analysis revealed that iron oxide nanoparticles were of high purity with crystalline cubic structure phases in nature. Besides, the average size of magnetite nanoparticles was observed to be 9–12 nm with mostly irregular shapes using a transmission electron microscope (TEM) and was supported by field emission scanning electron microscope (FESEM). Energy dispersive X-ray analysis shown that the elements iron (Fe) and oxygen (O) were present with atomic percentages of 33.29% and 66.71%, respectively. From the vibrating sample magnetometer (VSM) analysis it was proven that the nanoparticles exhibited superparamagnetic properties with a magnetization value of 73 emu/g and the results showed superparamagnetic behavior at room temperature, suggesting potential applications for a magnetic targeting drug delivery system.

## 1. Introduction

The emergence of nanoscale iron oxide nanoparticles with exceptional properties such as biocompatibility, stability in physiological environments, and size-dependent magnetic properties, superparamagnetism, high coercivity, high saturation magnetization and low toxicity [1,2], opens up a new window for medicine, biosensors, and drug delivery fields [2,3,4,5,6,7,8,9,10,11,12,13,14]. They are the only type of magnetic nanoparticles that are approved by food and drug administration (FDA) for clinical use [15]. Iron oxide nanoparticles have been realized by different synthesis routes through physical and chemical methods, which includes co-precipitation methods [16], micro-emulsion methods [17,18], sol-gel methods [19], solvothermal methods [20], thermal decomposition methods [21,22,23,24] and hydrothermal methods [25].

However, the chemical synthesis of iron oxide nanoparticles has drawbacks such as the usage of hazardous chemicals, formation of hazardous byproducts, contamination from precursor chemicals [26], highly reactive in nature and tend to form aggregates resulting in loss of reactivity [27,28], and the resulting iron oxide nanoparticles have limited application in biological systems due to low biocompatibility [29]. In addition, common methods like thermal decomposition and hydrothermal methods used to synthesize iron oxide nanoparticles also required high temperatures, high pressure, large amounts of toxic and expensive organic solvents [3].

Therefore, there is a growing need to develop clean, nontoxic, and environmentally friendly procedures for nanoparticle synthesis. Due to current sustainability concerns, the exploration of eco-friendly and green synthesis for the production of nanoparticles based on plant extracts has earned significant attention. Green synthesis are gaining importance by virtue of: (1) Clean and eco-friendly methods, as water is an environmentally friendly solvent [30]; (2) it is able to scale up [31]; (3) high energy and high pressure are not required, causing significant energy saving [32] and (4) finally, overall synthesis process is cost-effective as the active biological component able to act as a reducing and capping agent [30].

Great advances have been made for nanoparticle synthesis using a very wide range of biological resources like microorganisms and plants [33]. Plant extracts able to reduce the metal ions in a shorter time whereas microorganism-based methods require a longer time. The easy availability of plants in nature, make them more preferred biological resources than microbes [34,35,36,37]. Meanwhile, for microorganism-mediated nanoparticles, synthesis involves a lengthy process in maintaining cultures, the obligatory constraint of aseptic conditions, which requires trained staff, and these raise the scaling-up cost [38,39].

*Azadirachta indica*, which is a common plant known as Neem, belongs to the *Meliaceae* family and is found abundantly in Malaysia. It is known for its various applications especially its medicinal property [40]. The phytochemicals present in Neem are namely terpenoids and flavanones, which act as reducing as well as capping agent and helping in stabilizing nanoparticles. Extensive literature surveyed on the plant revealed that the major constituent of the *Azadirachta indica* are nimbin [41], nimbidin [42,43], ninbidol [44,45], gedunin [46], sodium nimbinate [47,48], quercetin [46,49], salannin [50] and Azadirachtin [41].

The co-precipitation method of iron salts has been reported to be the simplest and most efficient chemical pathway to synthesis magnetite nanoparticles [1,2]. The nanoparticles are usually prepared by a mixture of ferrous and ferric salts with alkaline medium. Here, we report a one-pot reaction, facile, safe and eco-friendly co-precipitation approach that utilizing *Azadiratcha Indica* extract is a non-toxic and naturally available material along with sodium hydroxide (NaOH) as the alkaline medium. The synthesized magnetite nanoparticles have been characterized by UV-VIS spectroscopy, Fourier transform infrared (FTIR), X-ray diffraction (XRD), transmission electron microscopy (TEM), field emission scanning electron microscope (FESEM) with energy dispersive X-ray spectrometer (EDX), and vibrating sample magnetometer (VSM).

## 2. Results and Discussions

### 2.1. Fourier Transform Infrared (FTIR) Spectroscopy

**Figure 1:**
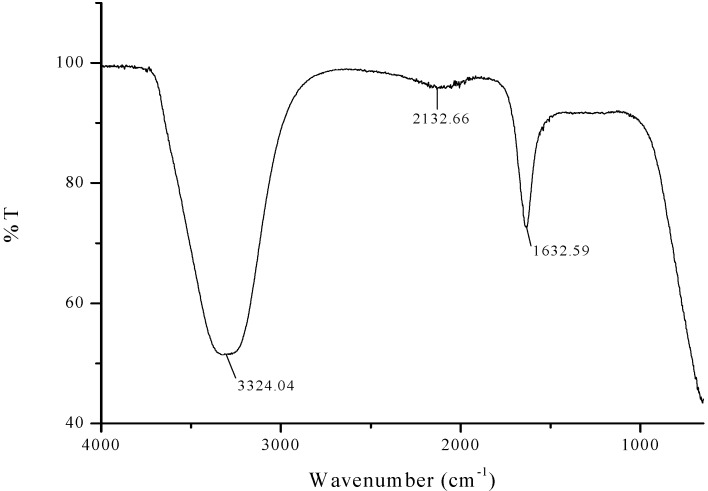
FTIR spectra of Neem leaf extract.

**Figure 2:**
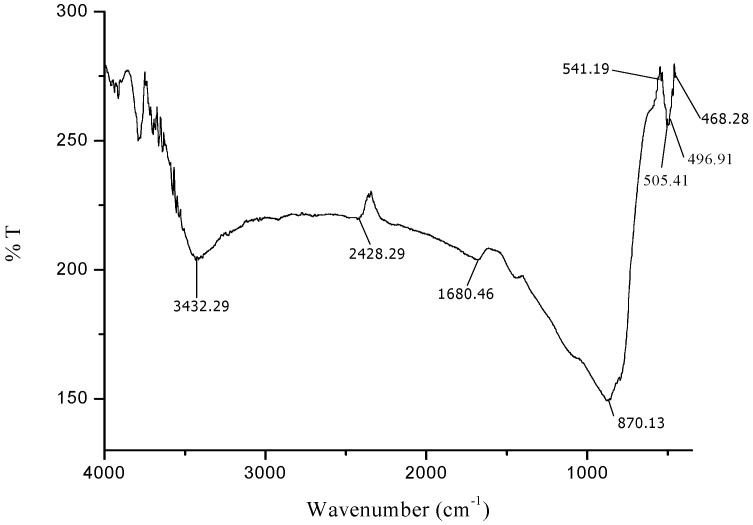
FTIR spectra of synthesized iron oxide nanoparticles.

FTIR analysis was carried out to identify the presence of flavanones and terpenoids in the *Azadirachta indica* leaf extract which are accountable for the stabilization of iron oxide nanoparticles. The representative FTIR spectra of pure *Azadirachta indica* leaf extract and the synthesized Fe_3_O_4_-NPs are manifested in Figure 1 and Figure 2. The strong stretching band appear around 3324 cm^−1^ (Figure 1) shows the presence of N-H stretching and bending vibration of amine group NH_2_ and OH the overlapping of the stretching vibration of attributed for water and phenolic group of *Azadirachta indica* leaf extract molecules. After reduction, the decreases in intensity at 3433 cm^−1^ imply the involvement of phenolic group of *Azadirachta indica* in the reduction process.

The FTIR spectra exhibited an adsorption peak at 1633 cm^−1^ (Figure 1), which can be attributed to amide C=O stretching indicating the presence of –COOH group in the *Azadirachta indica* leaf extract. The decreasing in intensity at 1680 cm^−1^ (Figure 2) signify the involvement of amide C=O stretching in the reduction process. Meanwhile, the adsorption peak at 2428 cm^−1^ (Figure 2) corresponds to alkyne group present in phytoconstituents of extracts. Hence, the presence of these functional groups validates that flavanones or terpenoids molecules were chemically bonded to the surface of Fe_3_O_4_-NPs. It is evident from the small shifts in the peak position (Figure 2) that there is strong interaction between Fe_3_O_4_-NPs with the flavanones or terpenoids of *Azadirachta indica* leaf extract molecules.

The appearance of new peaks (Figure 2) at 541 cm^−1^, 505 cm^−1^, 497 cm^−1^ and 468 cm^−1^ clearly indicate the presence of Fe-O stretching band of iron oxide nanoparticles [51,52]. This observation confirmed the formation of *Azadirachta indica* mediated Fe_3_O_4_-NPs in one-pot reaction. The FTIR data suggested that the reasonable mechanism of Fe_3_O_4_-NPs formation may be due to the reduction of iron ions that takes place together with the phenolic compounds in the *Azadirachta indica* leaf extract [53,54].

### 2.2. Ultraviolet-Visible (UV-VIS) Spectroscopy

**Figure 3:**
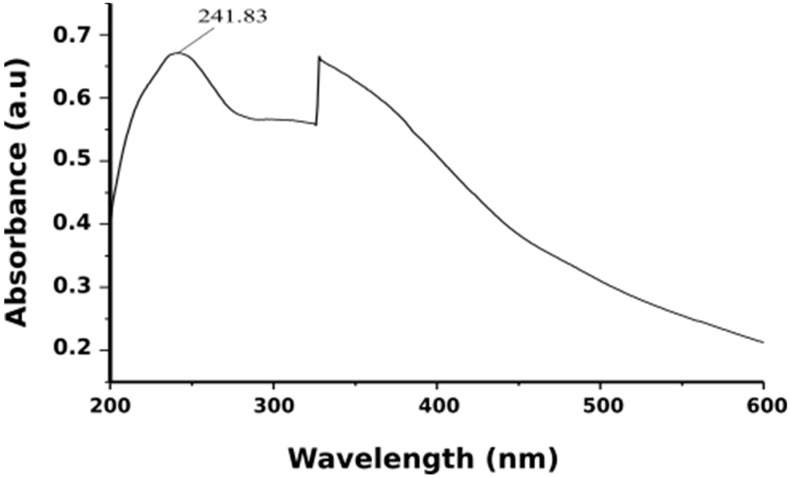
UV-visible absorption spectra of iron oxide nanoparticles.

When aqueous Neem leaf extract was added to iron salts it resulted in a color change from pale yellow to light orange and finally to black color. The transformation of the color of the solution was due to the presence of iron oxide nanoparticles formed by the reduction of iron salts. It was suggested that compounds like terpenoids and flavanones act as reducing agent when *Azadirachta indica* leaf extract was used [55]. It was noticed that the instantaneous color change took after about 10 min, thereafter no further color of the reaction mixture changed. This indicated that iron salts present in the reaction mixture had been reduced completely. Following this, the formation of Fe_3_O_4_-NPs was further confirmed by the UV-visible spectral analysis (Figure 3). The existence of a strong peak at around 242 nm regions was attributed to the excitation of surface plasmon vibrations in the iron oxide nanoparticles. This can be explained by the combined vibrations of free electrons of Fe_3_O_4_-NPs in resonance with light wave. Works from Hassan et al. (2015) and Devatha et al. (2016) had reported identical UV-VIS spectra for synthesis Fe_3_O_4_-NPs [56,57]. Thus, the Fe_3_O_4_-NPs can be synthesis within 10 min by this green method using non-toxic neem leaves make this synthesis method suitable for biomedical applications and biological studies.

### 2.3. Transmission Electron Microscopy (TEM)

**Figure 4:**
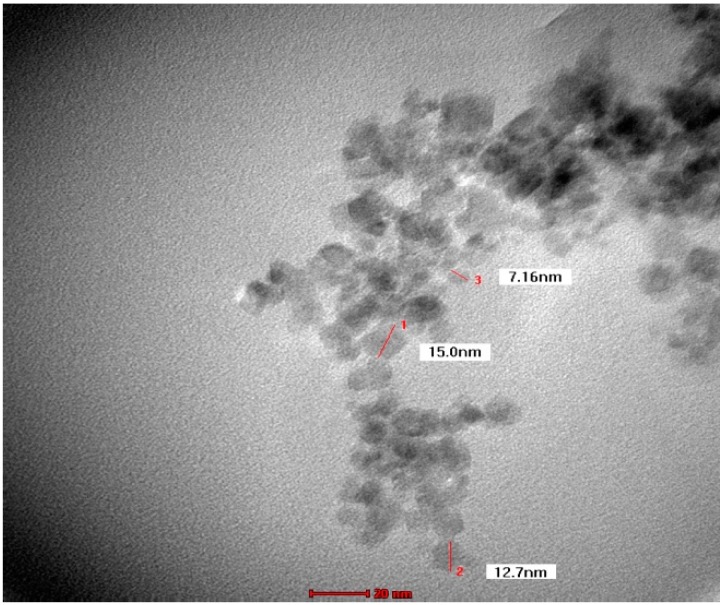
TEM micrograph of biosynthesized iron oxide nanoparticles.

TEM images recorded from drop coated grids of iron oxide nanoparticles are shown in Figure 4. Figure 4 shows that Fe_3_O_4_-NPs is found to be predominantly irregular in shape, and particle size ranging from 9 nm to 12 nm with some deviations. The obtained particle size coincidences with that calculated from Scherrer equation with average particle size of around 9.48 nm. The good correlation between particles sizes obtained from the Scherrer equation and TEM supports the highly crystalline structure of the iron nanoparticles as shown by XRD. Yew et al. (2016) and Mahdavi et al. (2013) reported the similar results for Fe_3_O_4_-NPs [58,59].

### 2.4. Field Emission Electron Microscopy (FESEM) and Energy Dispersive X-ray (EDX)

**Figure 5:**
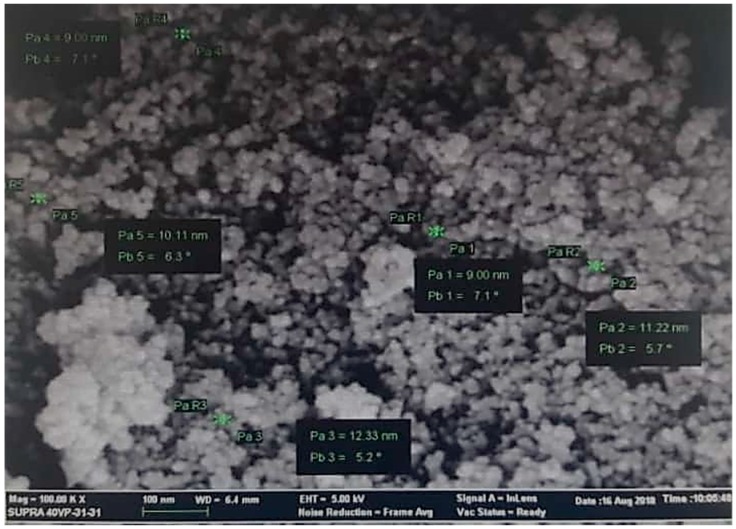
Field emission scanning electron microscopy (FESEM) micrograph of the biosynthesized iron oxide nanoparticles.

The surface morphology and elemental analysis of Fe_3_O_4_ nanoparticles were studied using FESEM coupled with EDX analysis. Figure 5 shows the FESEM image of the synthesized Fe_3_O_4_ nanoparticles consisted of nano-sized particles with a nearly spherical shape. It was observed that the nanoparticles formed were agglomerated with the particles and appear to adhere to each other, forming aggregate of particles, which result in irregular arrangements. The particles sizes measured about 9 to 12 nm, hence supporting the TEM result.

**Figure 6:**
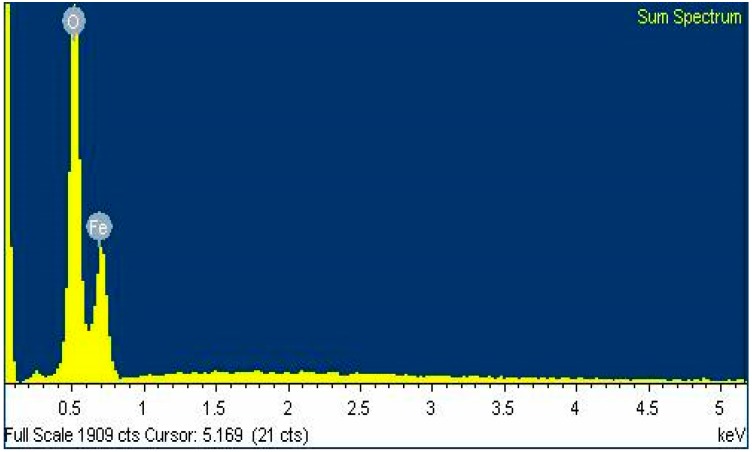
Energy dispersive X-ray spectroscopy (EDX) analysis spectra for Fe_3_O_4_ nanoparticles.

The presence of Fe (iron) and O (oxygen) with atomic percentage of 33.29% and 66.71% respectively was confirmed by EDX microanalysis (Figure 6). The presence of nanocrystalline elemental iron was confirmed by the distinctive peak, which was observed approximately at 0.6 keV. EDX spectra inferred the presence of elements Fe (iron) and O (oxygen) and thus confirmed the chemical composition of Fe_3_O_4_ nanoparticles.

### 2.5. Powder X-Ray Diffraction (PXRD)

**Figure 7:**
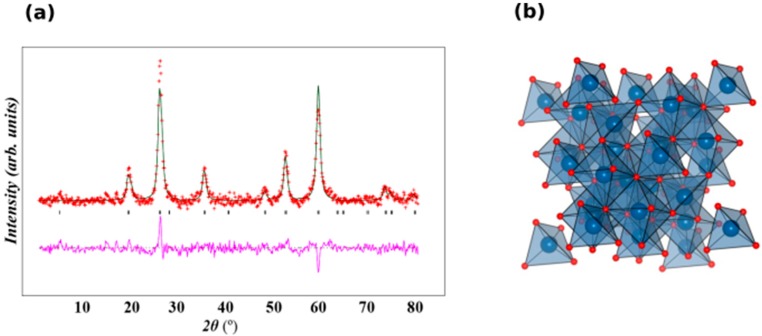
(**a**). XRD pattern with Rietveld refinement of Fe_3_O_4_. Crosses indicate the experimental data and the calculated profile is the black continuous line. The lowest curve shows the difference between experimental and calculated patterns. The black vertical bars indicate the expected reflection positions and (**b**) a polyhedral view of the Fe_3_O_4_ structure. Blue and red are occupied by Fe and O respectively.

The X-Ray powder diffraction measurement was performed at room temperature and a scan rate of 1° per minute in 0.013° steps, covering the 2θ angle from 15–80°. The XRD pattern of Fe_3_O_4_ showed that six characteristic diffraction peaks of Fe_3_O_4_ were observed at planes 2θ = (220) at 30.3° (311) at 35.6°, (400) at 43.3°, (422) at 53.2°, (511) at 57.1°, and (440) at 62.8°. The room temperature of the XRD pattern of Fe_3_O_4_ was plotted by Rietveld refinement using GSAS and EXPGUI software [60,61]. Sample were indexed to a single phase cubic structure with Fd3ms space group and all the diffraction peaks were in good agreement with the JCPDS file No.19-0629 (JCPDS). Lattice parameters were determined using Rietveld refinement as *a* = *b* = *c* = 8.3559 Å and the unit cell volume = 583.42 Å^3^. A lattice parameter value of 8.356 Å was reported for a similar synthesis of Fe_3_O_4_-NPs [62]. The observed lattice parameter was similar with that reported in the literature [63]. It was inferred that there were no other characteristic peaks detected in this pattern, indicating that purity of the synthesizes sample. Figure 7a and b show the Rietveld refinement of the XRD and the structural of Fe_3_O_4_. Average crystalline size (D) was determined using the Debye–Scherrer [64] equation: D = 0.94 λ/β cosθ, where λ is the wavelength of X-ray, β is the broadening of the peak at half maximum. The average crystallite size was found to be ~9.48 nm. The intense and sharp peaks undoubtedly revealed that Fe_3_O_4_-NPs formed by the reduction of iron ions using *Azadirachta indica* aqueous extract were highly crystalline in nature.

### 2.6. Magnetic Measurements

**Figure 8:**
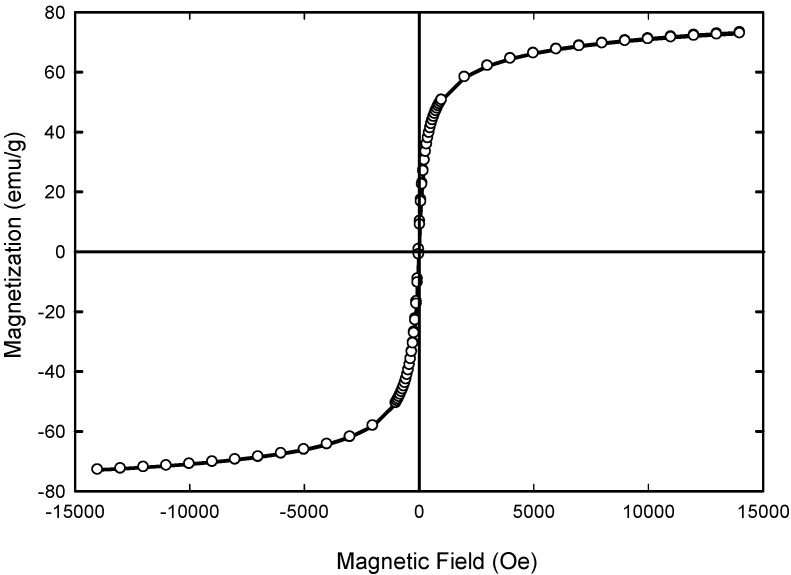
Magnetic hysteresis loop for the sample Fe_3_O_4_ at 300 K.

Figure 8 shows the magnetization curves as a function of applied external magnetic field. No hysteresis and remanence appeared indicating the superparamagnetic materials in nature. The maximum saturation magnetization (*Ms*) of Fe_3_O_4_ composite was 73.040 emu/g of Fe_3_O_4_ and the coercive field (Hc) were equal to 4 Oe. Previous study by Anbarasu [65] and co-researchers showed that when the resultant particle size was decreasing, the saturation magnetization (M_s_) of magnetic nanoparticles (MNPs) was reduced. The reduced saturation magnetization of Fe_3_O_4_ NPs compared with bulk magnetite (92 emu/g of Fe_3_O_4_) [63,66] is most likely due to the disordered spin layer at their surfaces [67]. Thus, when the size of the resultant particle size was decreasing, the ratio of disordered layer to the radius of the MNPs was significant. Surface spin disorder thus led to reduced *Ms* for smaller nanoparticles. Therefore, the way to enhance the *Ms* value was to increase the crystallize size of the magnetic particle. It has been reported that *Ms* of 7–22 emu/g is suitable for biomedical application [68,69]. Therefore, the level of *Ms* achieved for synthesized Fe_3_O_4_ nanoparticles is sufficient for biomedical applications.

### 2.7. Magnetic Behaviour and Colloidal Stability of Iron Oxide Nanoparticles

**Figure 9:**
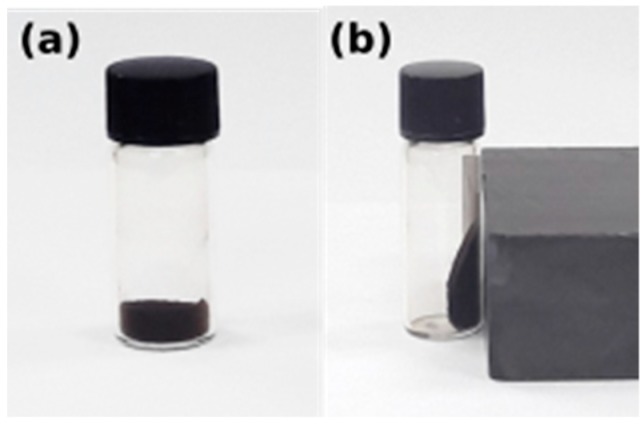
Iron oxide nanoparticles (**a**) without external magnetic field and (**b**) under external magnetic field.

Figure 9 shows the behavior of Fe_3_O_4_ nanoparticles before and after the external magnetic field. They discreted easily in double distilled water and also could be drawn from the solution to the side wall of the vial by an external magnet. The black suspended aqueous solution turned transparent within seconds when it was placed nearby, suggesting that the obtained Fe_3_O_4_ nanoparticles have an excellent magnetic responsive. No phase separation was observed for one month which suggests that the magnetite nanoparticles have good dispersion properties.

## 3. Materials and Methods

### 3.1. Materials

Iron (II) chloride tetrahydrate (FeCl_2_.4H_2_O) and iron (III) chloride hexahydrate (FeCl_3_.6H_2_O) were all purchased from Sigma Aldrich, St. Louis, MO, USA. NaOH pellets were obtained from MERCK KGaA (Darmstadt, Germany). All chemicals were of analytical grade and utilized without further purification.

### 3.2. Preparation of Azadirachta Indica (Neem Leaves) Extract

In this work, the healthy leaves of *Azadirachta indica* (neem leaves) were collected from Batu Caves, Selangor, Malaysia. The collected leaves were thoroughly washed several times with distilled water to remove dust particle and then air dried at room temperature to remove the remaining moisture. Then, the dried leaves were cut into small pieces and crushed into fine powder. Approximately, 5 g of finely grinded neem leaf powder was mixed with 100 mL sterile distilled water in a conical flask and the mixture was heated with boiling at constant temperature of 80 °C. Then, the mixture was left to cool at room temperature before vacuum-filtered through Whatman filter paper No. 1 to obtain plant extract. The green clear filtered solution of extract was stored at 4 °C for further use.

### 3.3. Biosynthesis of Iron Oxide (Fe_3_O_4_) Nanoparticles

Iron oxide nanoparticles were synthesized following a simple one-pot-two-step co-precipitation approach using *Azadirachta indica* (neem leaves) extract as the green reducing agent. In a typical procedure, 0.40 g iron (II) chloride tetrahydrate (FeCl_2_.4H_2_O) and 1.10 g of iron (III) chloride hexahydrate (FeCl_3_.6H_2_O) with 1:2 molar ratios were dissolved in 100 mL of sterile deionized water under nitrogen blanket. Following this, the resulting mixture was heated up to 80 °C under mild stirring for 10 min in oil bath to obtain homogeneous solution. Then, 5 mL of aqueous neem leaf extract was added slowly into the hot resulting mixture and subsequently 20 mL of sodium hydroxide (NaOH) was added into the reaction mixture drop by drop using burette under vigorous stirring for 30 min. The instantaneous black color appearance indicated the formation of Fe_3_O_4_-NPs. The resulting mixture was allowed to cool at room temperature. After 30 min, the solution was poured into a beaker and magnetic decantation was carried out in order to remove the supernatant. The intense black precipitate Fe_3_O_4_-NPs was then washed with 15 mL deionized water and centrifuged at 5000 rpm for 10 min in order to remove any residual salts. The supernatant was removed. The product was washed again with 10 mL of deionized water followed by centrifugation. The supernatant was removed again. The pellet was transferred to a vial and 10 mL deionized water was added. The obtained black powder was proceeded to freeze drying overnight and used for further characterizations.

### 3.4. Characterization

UV-visible spectral analysis was measured in aqueous solution by using Perkin Elmer UV-VIS spectrometer Lambda 35 (Perkin Elmer, Waltham, MA, USA) with a resolution of 1 nm between 200 and 600 nm to confirm the formation of Fe_3_O_4_ nanoparticles. The size and the morphology of the Fe_3_O_4_-NPs was investigated by means of a transmission electron microscopy (TEM) using Technai G2 20S Twin TEM, Eindhoven, Netherlands working at 200 kV. The samples were prepared by drop deposition of a diluted solution onto a carbon-coated copper grid. The grid was air-dried for a few hours prior to the TEM studies. X-ray powder diffraction (XRD) was used to analyze the structure and identify the phase purity of Fe_3_O_4_ compounds. The samples were placed on a flat plate while intensity data were collected as a function of the Bragg angle, θ, in the range 2θ = 15° to 80° with a step size of 0.013° using a PANanalytical X’pert PRO diffractometer in Bragg–Brentono geometry using Cu K_α_ radiation wavelength λ_α1_ = 1.5405 Å, λ_α2_ = 1.5443 Å. The structures were refined with the full-profile Rietveld method using GSAS [60] with the EXPGUI interface [61]. Fourier transform infrared (FTIR) spectra were collected using a Perkin Elmer Spectrum One FTIR spectrophotometer (Perkin Elmer) using the KBr pellet method with a range of 4000–400 cm^−1^. The surface morphology and atomic ratio of the Fe_3_O_4_-NPs was determined by using FESEM-EDX (ZEISS supra 40VP, Oberkochen, Germany). The magnetic properties of the prepared Fe_3_O_4_ was revealed using a vibrating sample magnetometer (VSM, Lake Shore 7404, McCorkle Boulevard, Westerville, OH, USA) at room temperature 300 K. The magnetization measurements, *M_s_* as a function of applied field (*H*) were measured under external magnetic fields up to ±14,000 Oe.

## 4. Conclusions

In this present study, we reported the successful use of *Azadirachta indica* as a one-pot green method for the synthesis of iron oxide nanoparticles. The color change, observed instantaneously suggested that the formation of black colored solution indicated the formation of iron oxide nanoparticles. The rapid reduction process proved the efficiency of *Azadirachta indica* extract as reducing and stabilizing agents. The XRD pattern showed the cubic crystal structure of iron oxide nanoparticles without any impurities. FTIR showed that the interactions that existed between *Azadirachta indica* and iron oxide nanoparticles. TEM showed the formation of Fe_3_O_4_ with an average size of 9–12 nm with irregular shape. Overall, the proposed green synthetic method was simple and eco-friendly, because it did not require any extra surfactants or reductants. Achievement of such rapid time scales for synthesis of iron oxide nanoparticles could be a competitive alternative to the conventional chemical protocols and a low cost candidate as reductant for synthesis of iron oxide nanoparticles, and thus, it has the potential to use in biomedical applications and will play an important role in magnetic targeting drug delivery in near future. From these studies, it can be inferred that Fe_3_O_4_-NPs biosynthesis may possibly be a gateway to our various health concerns.
